# Composite RAI, Malnutrition, and Anemia Model Superiorly Predicts 30-Day Morbidity and Mortality After Surgery for Adult Spinal Deformity

**DOI:** 10.3390/jcm14155379

**Published:** 2025-07-30

**Authors:** Aladine A. Elsamadicy, Paul Serrato, Shaila D. Ghanekar, Justice Hansen, Ethan D. L. Brown, Syed I. Khalid, Daniel Schneider, Sheng-fu Larry Lo, Daniel M. Sciubba

**Affiliations:** 1 Department of Neurosurgery, Zucker School of Medicine at Hofstra, Long Island Jewish Medical Center and North Shore University Hospital, Northwell Health, Manhasset, NY 11030, USA; ebrown35@northwell.edu (E.D.L.B.); dschneider5@northwell.edu (D.S.); larrylo@northwell.edu (S.-f.L.L.); dsciubba1@northwell.edu (D.M.S.); 2 Department of Neurosurgery, Yale University School of Medicine, New Haven, CT 06510, USA; paul.serrato@yale.edu (P.S.); shaila.ghanekar@yale.edu (S.D.G.); justice.hansen@yale.edu (J.H.); 3 Department of Neurosurgery, University of Illinois at Chicago, Chicago, IL 60612, USA; syed.khalid@me.com

**Keywords:** adult spinal deformity, frailty, malnutrition, anemia, risk analysis index (RAI), morbidity

## Abstract

**Background/Objective:** This study examines the composite influence of frailty, malnutrition, and anemia on postoperative outcomes for patients with adult spinal deformity (ASD). **Methods:** In this retrospective cohort study using the 2011–2022 NSQIP database, we utilized CPT and ICD codes to identify ASD patients who underwent PSF. Subjects were stratified based on frailty status. Frail patients were then classified according to malnutrition and anemia status. Frailty was determined using the revised risk analysis index (RAI-rev). Our primary outcomes were extended length of stay (LOS), non-routine discharge (NRD), 30-day adverse events (AE), and 30-day mortality. For each outcome, we fitted four nested multivariable logistic regression models (RAI-rev + anemia + malnutrition, RAI-rev + anemia, RAI-rev + malnutrition, and RAI-rev alone) and compared the incremental discrimination of each model using receiver operating characteristic (ROC) analysis. **Results:** Of 3639 patients, 460 were frail alone, 266 were frail + anemic, 37 were frail + malnourished, 121 were frail + anemic + malnourished, and 2755 were not frail. RAI-rev (aOR: 1.84, 95% CI: 1.45–2.35), anemia (aOR: 1.84, 95% CI: 1.45–2.35), and malnourishment (aOR: 2.34, 95% CI: 1.69–3.24) were independent predictors of extended LOS. RAI-rev (aOR: 1.07, 95% CI: 1.04–1.11) and anemia (aOR: 2.09, 95% CI: 1.66–2.61) were associated with an increased risk of 30-day AEs. RAI-rev and malnutrition were independent predictors of NRD (RAI-rev: aOR: 1.11, 95% CI: 1.06–1.16; Malnutrition: aOR: 1.57, 95% CI: 1.08–2.29) and 30-day mortality (RAI-rev: aOR: 1.10, 95% CI: 1.04–1.17; Malnutrition: aOR: 3.79, 95% CI: 1.24–11.60). Based on ROC analysis, RAI-rev + anemic + malnourished was a superior predictor of LOS and 30-day AEs (both *p* < 0.001). Compared to RAI-rev, RAI-rev + anemic superiorly predicted LOS and 30-day AEs, and RAI-rev + malnutrition superiorly predicted LOS (all *p* < 0.001). **Conclusions:** Our results reveal RAI-rev combined with malnutrition and anemia superiorly predicts 30-day AEs and LOS in postoperative ASD patients. Future studies should investigate the feasibility and efficacy of these models for perioperative risk stratification and optimized recovery planning to improve outcomes for ASD patients.

## 1. Introduction

The past decade has seen an exponential rise in US healthcare costs, with total spending reaching USD 4.9 trillion [1]. Efforts to mitigate expenditures without sacrificing care quality have sparked the development of value-based care protocols [2,3]. Various outcome measures, including hospital length of stay (LOS), discharge disposition, and unplanned readmission, have emerged as proxies for evaluating these protocols [4,5]. In spine surgery patients, extended LOS and unplanned readmission have been linked with increased costs, adverse events (AEs), and mortality [6,7]. One subgroup within spine surgery that would benefit from targeted initiatives to optimize patient outcomes and cost efficiency is adult spinal deformity (ASD) patients. In recent years, there has been an increasing prevalence of ASD [8]—affecting 30–70% of adults older than 60 [9]—and a resulting increase in spinal interventions [8]. These corrective surgical procedures have been associated with higher complication rates, thus increasing the burden on the healthcare system [10,11]. Attempts to optimize perioperative care and decrease costs have sparked an examination of potential risk factors that may influence ASD outcomes.

As the population’s median age increases, one risk factor that has been noted to influence outcomes is frailty. Frailty—decreased adaptability to stressors and lessened reserve of physiologic systems [12]—has been associated with increased mortality, nonroutine discharge (NRD), LOS, readmission, and reoperation [6,13,14,15]. There are various validated indexes, including the Charlson Comorbidity Index [16], the modified frailty index [17], and the recently developed risk analysis index (RAI) [18], which incorporate several comorbidities, such as congestive heart failure (CHF), diabetes, and renal disease, to quantify frailty. In addition to these chronic conditions, frailty often co-occurs with other risk factors, such as anemia [19] and malnutrition [20,21], for adverse surgical outcomes. Despite the increased prevalence of anemia and malnutrition in the setting of frailty, there is a paucity of studies evaluating the combined impact of frailty, anemia, and malnutrition on postoperative ASD outcomes.

This study aims to examine the interplay of frailty, anemia, and malnutrition and their influence on morbidity and mortality in ASD patients undergoing posterior spinal fusion (PSF).

## 2. Materials and Methods

### 2.1. Data Source

The American College of Surgeons (ACS) National Surgical Quality Improvement Program (NSQIP) is a prospectively collected, peer-controlled database developed for the examination of 30-day risk-adjusted surgical outcomes. Trained personnel abstracted data based on patient records. All data were deidentified, so institutional review board evaluation of this study was not required.

### 2.2. Patient Cohort

**Figure 1:**
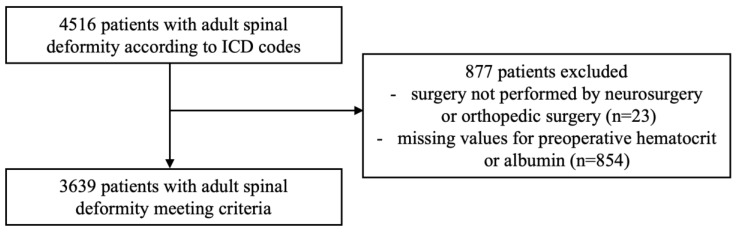
Flow diagram for inclusion and exclusion criteria.

We searched the NSQIP Participant Use Data Files from 2011–2022 for all patients ≥18 years of age who underwent corrective surgery for adult spinal deformity using Current Procedural Terminology (CPT) codes for long fusion-hardware constructs (≥7-level). These CPT codes were 22800, 22802, 22804, 22808, 22810, 22812, 22818, 22819, 22843, 22844, 22846, or 22847. In addition, patients who underwent <7-level fusion (CPT codes 22842 or 22845) were also included if they had a concurrent International Classification of Diseases (ICD) code for spinal deformity, Supplementary Table S1. This approach has been used in the literature to examine this population. [22] Patients who underwent a <7-level spinal fusion without concomitant ICD codes for spinal deformity were excluded. Only procedures performed by neurosurgeons or orthopedic surgeons were included, Figure 1. Study size included all patients meeting inclusion criteria within the timeframe.

### 2.3. Exposure Definitions

The RAI is a frailty assessment tool that integrates multiple domains, including comorbidities, functional status, nutritional status, and cognitive function. It was recalibrated and validated for surgical populations using large national databases, resulting in the RAI-rev, which improves mortality prediction [23]. As the NSQIP database does not provide all the variables necessary for a complete RAI-rev calculation, we derived the RAI-rev using the available dataset variables as performed in the literature [24] and detailed in Supplementary Table S2. Specifically, we substituted weight loss as a proxy for poor appetite and omitted cognitive decline. The adapted cumulative RAI-rev score ranged from 0 to 78. We utilized RAI-rev to determine frailty in our cohort, classifying participants with scores exceeding 20 as frail, following the criteria set by Conlon et al. [24]. Furthermore, we stratified frail patients based on the presence of anemia and malnutrition. Anemia was defined by preoperative hematocrit levels under 41 for males and under 36 for females. Malnutrition was defined as preoperative serum albumin <3.5 g/dL, a well-established threshold for hypoalbuminemia and nutritional risk [25,26].

### 2.4. Outcome Definitions

Demographic variables in our analysis included age, sex, race/ethnicity, and body mass index (BMI). Comorbidities included American Society of Anesthesiologists (ASA) grade, diabetes mellitus, hypertension, CHF, chronic obstructive pulmonary disease (COPD), dependent functional status, disseminated cancer, electrolyte abnormalities (defined as preoperative sodium <135 mEq/L or >145 mEq/L), smoking, chronic steroid use, and bleeding disorders. Postoperative AEs occurred within the first 30 days after surgery as provided by NSQIP. These events were categorized into surgical and medical AEs. Surgical AEs included superficial surgical site infection (SSI), organ space SSI, deep SSI, and wound dehiscence. We examined Medical AEs including pneumonia (PNA), need for mechanical ventilation, unplanned reintubation, pulmonary embolism (PE), deep vein thrombosis (DVT), myocardial infarction (MI), *C. diff* colitis, urinary tract infection (UTI), renal insufficiency, acute renal failure (ARF), systemic sepsis, septic shock, and postoperative red blood cell (RBC) transfusion. AEs were also classified by severity: minor (MAE) and severe (SAE). MAEs included superficial SSI, PNA, renal insufficiency, and UTI, while SAEs included all other AEs. In addition, we examined healthcare utilization outcomes—total operation time (hours), rates of readmission, and incidence of reoperation. Primary outcomes included prolonged LOS (8 days or LOS greater than the 75th percentile for the entire cohort), NRD (discharge to a location other than home or permanent residence), 30-day AEs, and 30-day mortality. We dichotomized LOS using this threshold for ease of interpretability and to avoid issues with skewness and overdispersion that would limit other analytic approaches. Mortality was established using discharge disposition, end-of-life care markers, and death date. Bias was minimized by excluding missing data and with a large-sized sample representative of the larger population.

### 2.5. Statistical Analysis

The study population was stratified based on frailty status, with frail patients being further categorized according to malnutrition and anemia status. Continuous variables were summarized by mean and SD and categorical variables using frequencies and percentages. Group comparisons utilized ANOVA for normally distributed continuous data, Kruskal–Wallis for non-normal data, and chi-squared or Fisher’s exact tests for categorical data. To evaluate predictors of the study’s primary outcomes, multivariable logistic regression models were created, incorporating RAI-rev, anemia, and malnutrition into each model; we developed four nested logistic-regression models that differed only in the anemia-nutrition variables they contained: (1) RAI-rev + anemia + malnutrition (full model), (2) RAI-rev + anemia, (3) RAI-rev + malnutrition, and (4) RAI-rev alone. For each outcome, the nested models were adjusted for the same covariates, which were determined by considering clinically relevant preoperative and operative variables. Next, the Akaike Information Criterion (AIC) were employed to refine each model in a stepwise fashion, utilizing backward elimination for identifying and retaining the most significant predictors. Adjusted odds ratios (ORs) with 95% confidence intervals were subsequently calculated.

For each outcome, receiver operating characteristic (ROC) curves were created to assess each of the four models’ predictive performance. DeLong tests were then used to compare the area under the curve (AUC), and 95% CIs to quantify the incremental discrimination. Adjusted regression coefficients and corresponding AUCs can be found in the tables and figures, respectively. All tests were two-sided, and the significance was set to *p* ≤ 0.05. Observations with missing data were excluded (Supplementary Table S3). RStudio v4.4.2 (R Foundation for Statistical Computing, Boston, MA, USA) was utilized for statistical analyses.

## 3. Results

### 3.1. Patient Demographics and Comorbidities

Of the 3639 patients identified, 460 (12.7%) were frail alone (F), 266 (7.3%) were frail + anemic (FA), 37 (1.0%) were frail + malnourished (FM), 121 (3.3%) were frail + anemic + malnourished (FAM), and 2755 (75.7%) were not frail (NF). Age (*p* < 0.001), sex (*p* < 0.001), and racial/ethnic (*p* = 0.005) makeup varied significantly, Table 1. NF patients had a lower BMI than patients in the other cohorts (*p* < 0.001), Table 1. The FAM cohort had the highest proportion of patients with an ASA grade of ≥4 (*p* < 0.001), Table 1.

### 3.2. 30-Day Complications and Hospital Outcomes

Frail patients with anemia, malnutrition, or both consistently had worse outcomes than patients in the F cohort. FAM patients had the highest frequency of any AE, reintubation, ventilator requirement, and septic shock (all *p* < 0.001), Table 2. The FA cohort had the highest incidence of PE (*p* < 0.001), renal insufficiency (*p* = 0.004), UTI (*p* = 0.001), DVT (*p* = 0.032), *C. diff* colitis (*p* = 0.039), and postoperative RBC transfusion (*p* < 0.001), Table 2. NRD (*p* < 0.001) was more frequent in FM patients whereas the longest mean LOS (*p* < 0.001) and highest frequencies of readmission (*p* = 0.010) and mortality (*p* < 0.001), Table 2, occurred in FAM patients. Conversely, the NF cohort had the longest total operation time (*p* = 0.017), Table 2. The predominance of worse outcomes among frail patients with malnutrition, anemia, or both could indicate an increased risk conferred by the combination of these factors compared to frailty alone.

### 3.3. Multivariable Logistic Regression and ROC Analysis Comparing Predictive Models

#### 3.3.1. Extended LOS

**Figure 2:**
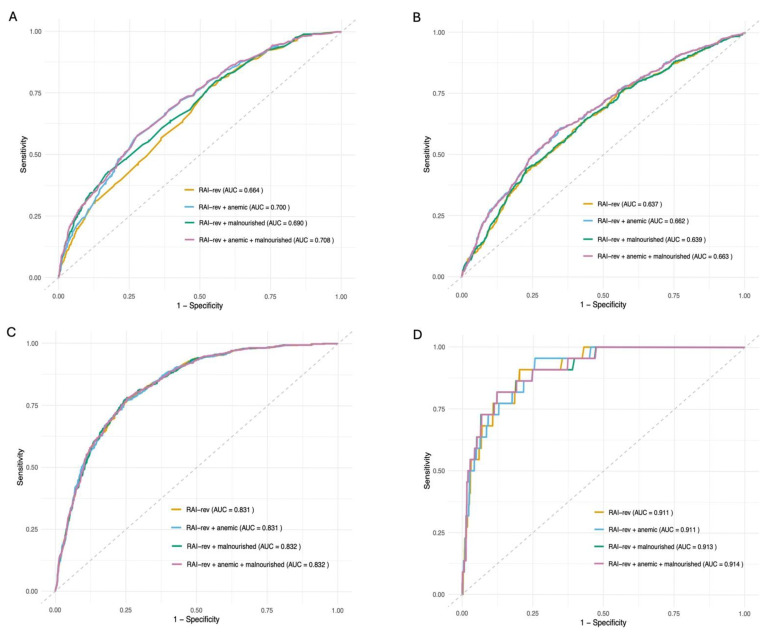
Comparing model performance for predicting (**A**) extended hospital length of stay (LOS), (**B**) 30-day adverse events (AEs), (**C**) non-routine discharge (NRD), and (**D**) 30-day mortality using area under the receiver operating characteristic (AUROC) curves. (**A**) *p*-values: RAI-rev + anemic vs. RAI rev: *p* < 0.001; RAI-rev + malnourished vs. RAI-rev: *p* < 0.001; RAI-rev + anemic + malnourished: *p* < 0.001; Models adjusted for sex, ASA classification, dependent functional status, and bleeding disorder. (**B**) *p* values: RAI-rev + anemic vs. RAI rev: *p* < 0.001; RAI-rev + malnourished vs. RAI-rev: *p* = 0.403; RAI-rev + anemic + malnourished: *p* < 0.001; Models adjusted for age, sex, race, ASA classification, diabetes mellitus, disseminated cancer, and smoking. (**C**) *p* values: RAI-rev + anemic vs. RAI rev: *p* = 0.724; RAI-rev + malnourished vs. RAI-rev: *p* = 0.609; RAI-rev + anemic + malnourished: *p* = 0.522; Models adjusted for age, sex, BMI, hypertension, diabetes mellitus, COPD, disseminated cancer, SAE, hospital length of stay, and operation time. (**D**) *p* values: RAI-rev + anemic vs. RAI rev: *p* = 0.996; RAI-rev + malnourished vs. RAI-rev: *p* = 0.810; RAI-rev + anemic + malnourished: *p* = 0.760; Models adjusted for age, electrolyte abnormality, smoking, preoperative steroids, bleeding disorder, MAE, and SAE.

Based on multivariable analysis, RAI-rev-defined frailty (aOR: 1.03, 95% CI: 1.01–1.04), anemia (aOR: 1.84, 95% CI: 1.45–2.35), and malnourishment (aOR: 2.34, 95% CI: 1.69–3.24), Table 3, independently predicted extended LOS. Based on ROC analysis, RAI-rev + anemic + malnourished had the highest AUC of 0.708, indicating moderate discrimination. In other words, given a randomly selected pair of patients, the model has a 71% chance of assigning a higher risk score to the patient with extended LOS. This was followed by RAI-rev + anemic with an AUC of 0.700, RAI-rev + malnourished with an AUC of 0.690, and RAI-rev with an AUC of 0.664, Figure 2A. Compared to RAI-rev, RAI-rev + anemic, RAI-rev + malnourished, and RAI-rev + anemic + malnourished (all *p* < 0.001) superiorly predicted extended LOS, Figure 2A.

#### 3.3.2. 30-Day Adverse Events

Based on multivariable analysis, risk for 30-day AEs was associated with RAI-rev-defined frailty (aOR: 1.07, 95% CI: 1.04–1.11) and anemia (aOR: 2.09, 95% CI: 1.66–2.61), Table 3. Based on ROC analysis, RAI-rev + anemic + malnourished had an AUC of 0.663, RAI-rev + anemic an AUC of 0.662, RAI-rev + malnourished an AUC of 0.639, and RAI-rev an AUC of 0.637, Figure 2B. RAI-rev + anemic + malnourished (*p* < 0.001) and RAI-rev + anemic (*p* < 0.001) superiorly predicted 30-day AEs compared to RAI-rev, Figure 2B.

#### 3.3.3. Non-Routine Discharge

Based on multivariable analysis, RAI-rev-defined frailty (aOR: 1.11, 95% CI: 1.06–1.16) and malnourishment (aOR: 1.57, 95% CI: 1.08–2.29), Table 4, independently predicted NRD. Based on ROC analysis, RAI-rev + anemic + malnourished and RAI-rev + malnourished had identical AUCs of 0.832, and RAI-rev + anemic and RAI-rev had identical AUCs of 0.831, Figure 2C. No significant differences were noted in the models for predicting NRD, Figure 2C.

#### 3.3.4. 30-Day Mortality

Based on multivariable analysis, 30-day mortality was independently predicted by RAI-rev-defined frailty (aOR: 1.10, 95% CI: 1.04–1.17) and malnourishment (aOR: 3.79, 95% CI: 1.24–11.60), Table 4. Based on ROC analysis, RAI-rev + anemic + malnourished had an AUC of 0.914, RAI-rev + malnourished an AUC of 0.913, and RAI-rev + anemic and RAI-rev AUC of 0.911, Figure 2D. No significant differences were noted in the models for predicting 30-day mortality, Figure 2D.

## 4. Discussion

In our study of 3639 ASD patients undergoing PSF, frail patients with anemia, malnutrition, or both had a higher frequency of worse outcomes, including extended LOS, NRD, readmission, and 30-day mortality. RAI-rev-defined frailty, anemia, and malnourishment independently predicted extended LOS. RAI-rev-defined frailty and anemia were risk factors for 30-day AEs while RAI-rev-defined frailty and malnutrition were associated with NRD and 30-day mortality. While the RAI-rev + anemic + malnourished and RAI-rev + anemic models were superior predictors of LOS and 30-day AEs, RAI-rev + malnutrition only outperformed RAI-rev in predicting LOS.

The impact of frailty on outcomes in postoperative ASD patients has been previously studied. In a retrospective cohort study of 1001 ASD patients, Leven et al. found that increased frailty independently predicted AEs, mortality, and unplanned reoperation [27]. In addition, in a multicenter prospective database study of 417 ASD patients, Miller et al. demonstrated increased LOS and higher rates of major complications and reoperation in frailer patients [28]. Furthermore, Baek et al., in a systematic review and meta-analysis of 474,651 degenerative spine surgery patients, demonstrated frailty to be a robust predictor of poor outcomes, including mortality, AEs, NRD, and extended LOS [29]. Similarly, our study showed RAI-rev-defined frailty to be an independent predictor of extended LOS, NRD, 30-day AEs, and mortality.

Prior investigations into anemia’s effect on ASD outcomes have demonstrated significant effects. In a retrospective cohort study of 2173 ASD patients undergoing PSF, Mo et al. found that increasing anemia severity was associated with higher odds of extended LOS, transfusion, organ space infection, and mortality [30]. Jung et al., in another retrospective database study of ASD patients, reported that patients with anemia had a higher rate of AEs, including transfusions, wound complications, and overall surgical complications [31]. In a retrospective cohort study of 473 elective posterior cervical fusion patients, Phan et al. demonstrated an increased rate of readmission, reoperation, and mortality, as well as AEs and extended LOS, in anemic patients [32]. Analogously, our results showed anemia to be an independent predictor of extended LOS and 30-day AEs.

Malnutrition has been assessed as a risk factor for adverse outcomes in ASD. In a retrospective cohort study of 303 ASD patients, Wang et al. reported a greater incidence of postoperative complications in malnourished patients [33]. Similarly, Oe et al. noted that malnutrition was an independent predictor of postoperative complications in a retrospective cohort study of 285 ASD patients [34]. In addition, a retrospective cohort study of 136 spine fusion patients by Adogwa et al. demonstrated increased complications in malnourished patients and noted malnutrition to be an independent predictor of AEs [35]. Comparatively, in our study, malnutrition independently predicted extended LOS and NRD.

While evidence demonstrating the effect of frailty, anemia, and malnutrition on postoperative outcomes is robust, few studies have evaluated the combined influence of these factors. In a retrospective cohort study of 923 digestive tract surgery patients, Li et al. noted an increased risk of adverse outcomes when combining frailty, malnutrition, and anemia [36]. In another retrospective cohort study of patients undergoing PSF, Han et al. reported that a combined frailty and malnutrition model outperformed the individual risk factors in predicting AEs [37]. Similarly, Camino-Willhuber et al., in a retrospective cohort study of 69,519 spine surgery patients, found a higher risk of complications, readmission, reoperation, and mortality among malnourished frail patients compared to nourished frail patients [38]. Our results demonstrated that models combining RAI-rev-defined frailty with malnutrition and anemia were superior predictors of postoperative outcomes in ASD patients. However, it is important to note that our model for mortality was limited by a low number of events, which may have led to model instability. Although we used AIC-guided stepwise selection to mitigate overfitting, the small event count restricted the model’s discriminatory power, as reflected by the minimal differences in AUCs across nested models. As such, findings related to mortality should be interpreted with caution.

Understanding the interplay of these risk factors will promote the development and implementation of evidence-based care pathways. For example, Enhanced Recovery After Surgery (ERAS) protocols are perioperative management guidelines that have been reported to optimize patient outcomes and decrease expenditures in spine surgery [39]. While studies assessing ERAS protocols in frail patients are limited, existing results are promising. In a retrospective cohort study of 32 frail TLIF patients, Porche et al. found that ERAS protocols shortened LOS [40]. In another retrospective cohort study of 128 frail lumbar fusion surgery patients, Cui et al. reported ERAS protocols decreased LOS and functional recovery time [41]. In terms of nutrition intervention, Chen et al. noted shorter LOS and accelerated return to normal diet with ERAS nutrition protocols [42]. Similarly, Saleh et al., in a randomized controlled trial of spine surgery patients, noted nutrition supplementation decreased complications [43]. Concerning anemia, studies have shown that preoperative treatment decreases LOS and the need for postoperative transfusions [44,45]. While these results demonstrate the utility of ERAS protocols and other focused interventions, further research is essential to better assess their efficacy in mitigating the influence of frailty, malnutrition, and anemia in spine surgery. Furthermore, considering the demonstrated efficacy of ERAS protocols, it is pertinent for future studies to evaluate the feasibility and efficacy of incorporating malnutrition and anemia screening into the existing ERAS clinical workflow.

The aforementioned studies present strong evidence for the use of perioperative risk stratification protocols and patient optimization for individual factors, like frailty, anemia, and malnutrition. Considering the results of our study demonstrating the composite model’s superior predictive capacity for 30-day AEs and extended LOS, protocols incorporating this composite model could have greater utility in identifying higher-risk patients. This composite model could be incorporated into pre-existing workflows, such as ERAS, and then run through an electronic medical database to automatically flag patients meeting high-risk criteria. Identifying high-risk patients who could benefit from optimization, such as nutrition supplementation, blood transfusions, or special frailty considerations, may enhance surgical planning, decision-making on the timing of surgery, and the utility of intraoperative measures for patients with multiple risk factors for worse outcomes. In addition, early recognition of higher-risk patients will facilitate a more individualized approach to care planning, such as at the level of pain control and anesthesia [46]. Future studies should work to assess the feasibility and cost-efficacy of implementing perioperative screening utilizing electronic medical record systems, as well as evaluate how these protocols can help to ameliorate outcomes in high-risk patients.

Our study has limitations that warrant cautious interpretation of our findings. Using the NSQIP database introduces potential coding errors, misclassification, and reporting biases. This study relied on procedural coding used in the literature to identify ASD cases, which may still be susceptible to misclassification. Inaccurate or inconsistent coding of frailty, anemia, or malnutrition could lead to misidentification of patient status, which may result in either an overestimation or underestimation of associations. Although we adjusted for numerous demographic and clinical variables in our multivariable models, residual confounding remains a concern due to unmeasured factors such as socioeconomic status (health insurance status, income), surgeon expertise, perioperative protocols, length of fusion, number of instrumented levels, and preoperative management of anemia and malnutrition. These unmeasured variables might create bias in either direction. The study’s retrospective design precludes our ability to draw causal inferences and introduces potential selection bias through non-randomized assignments. This bias may result in overestimation of effects if more severe patients are preferentially classified as frail or may lead to underestimation if protective factors are underrepresented. This adaptation of RAI-rev has not been externally validated but has been used in previous studies in the literature using NSQIP data. Furthermore, the substitution of variables may lead to misclassification which we were unable to assess. Finally, the NSQIP database only provides outcomes within a 30-day postoperative window, which may not fully capture long-term complications and mortality. Despite these limitations, to our knowledge, this is the only large-scale study assessing the combined role of frailty, malnutrition, and anemia in predicting surgical outcomes in ASD.

## 5. Conclusions

The results of our study reveal that RAI-rev-defined frailty has superior predictive capacity for 30-day AEs and LOS when combined with anemia and malnutrition. Future studies should further evaluate the predictive capacity of this composite model as well as assess its utility in perioperative risk stratification to inform prehabilitation measures and shared decision-making for high-risk ASD patients.

## Figures and Tables

**Table 1:** Patient demographics and comorbidities by health condition combination.

Variables	Frail Alone (n = 460)	Frail + Anemic (n = 266)	Frail + Malnourished (n = 37)	Frail + Anemic + Malnourished (n = 121)	Not Frail (n = 2755)	*p*-Value
Age (years), mean (SD)	70.94 (7.69)	69.85 (8.05)	73.08 (9.24)	70.47 (8.75)	45.50 (17.48)	<0.001 *
Female, n (%)	245 (53.3)	82 (30.8)	21 (56.8)	57 (47.1)	1992 (72.3)	<0.001 *
Race/Ethnicity, n (%)						0.005 *
NHW	373 (85.9)	186 (76.9)	18 (64.3)	85 (76.6)	1866 (80.6)	
NHB	27 (6.2)	26 (10.7)	3 (10.7)	13 (11.7)	242 (10.4)	
Hispanic	16 (3.7)	21 (8.7)	5 (17.9)	6 (5.4)	127 (5.5)	
Other	18 (4.1)	9 (3.7)	2 (7.1)	7 (6.3)	81 (3.5)	
BMI (kg/m^2^), mean (SD)	29.33 (6.02)	29.07 (6.84)	29.17 (6.70)	28.10 (6.43)	27.49 (7.25)	<0.001 *
ASA, n (%)						<0.001 *
1	2 (0.4)	0 (0.0)	0 (0.0)	0 (0.0)	186 (6.8)	
2	118 (25.7)	38 (14.3)	7 (18.9)	16 (13.2)	1190 (43.3)	
3	328 (71.3)	199 (75.1)	24 (64.9)	75 (62.0)	1302 (47.3)	
≥4	12 (2.6)	28 (10.6)	6 (16.2)	30 (24.8)	72 (2.6)	
Hypertension, n (%)	324 (70.4)	193 (72.6)	23 (62.2)	86 (71.1)	879 (31.9)	<0.001 *
Diabetes mellitus, n (%)	79 (17.2)	66 (24.8)	12 (32.4)	35 (28.9)	244 (8.9)	<0.001 *
COPD, n (%)	28 (6.1)	19 (7.1)	3 (8.1)	9 (7.4)	82 (3.0)	<0.001 *
CHF, n (%)	9 (2.0)	9 (3.4)	2 (5.4)	2 (1.7)	5 (0.2)	<0.001 *
Dependent functional status, n (%)	33 (7.2)	31 (11.7)	3 (8.1)	34 (28.1)	95 (3.5)	<0.001 *
Disseminated cancer, n (%)	14 (3.0)	25 (9.4)	7 (18.9)	12 (9.9)	0 (0.0)	<0.001 *
Electrolyte abnormality, n (%)	28 (6.1)	25 (9.5)	5 (13.5)	26 (21.5)	143 (5.9)	<0.001 *
Smoking, n (%)	33 (7.2)	46 (17.3)	7 (18.9)	18 (14.9)	540 (19.6)	<0.001 *
Pre-operative steroids, n (%)	24 (5.2)	19 (7.1)	2 (5.4)	21 (17.4)	111 (4.0)	<0.001 *
Bleeding disorder, n (%)	10 (2.2)	17 (6.4)	2 (5.4)	4 (3.3)	43 (1.6)	<0.001 *

SD: standard deviation; NHW: non-Hispanic White; NHB: non-Hispanic Black; BMI: body mass index; ASA: American Society of Anesthesiologists classification; COPD: chronic obstructive pulmonary disease; CHF: congestive heart failure; * Statistically significant, unadjusted *p*-value.

**Table 2:** Rates of postoperative outcomes by health condition combination.

Variables	Frail Alone (n = 460)	Frail + Anemic (n = 266)	Frail + Malnourished (n = 37)	Frail + Anemic + Malnourished (n = 121)	Not Frail (n = 2755)	*p*-Value
Any AE, n (%)	213 (46.3)	161 (60.5)	16 (43.2)	75 (62.0)	1259 (45.7)	<0.001 *
Surgical AEs, n (%)						
Superficial SSI	5 (1.1)	8 (3.0)	0 (0.0)	2 (1.7)	37 (1.3)	0.214
Deep SSI	4 (0.9)	2 (0.8)	1 (2.7)	1 (0.8)	25 (0.9)	0.842
Organ space SSI	4 (0.9)	5 (1.9)	0 (0.0)	3 (2.5)	33 (1.2)	0.488
Wound dehiscence	2 (0.4)	3 (1.1)	0 (0.0)	1 (0.8)	23 (0.8)	0.830
Medical AEs, n (%)						
PNA	13 (2.8)	14 (5.3)	2 (5.4)	5 (4.1)	66 (2.4)	0.051
Reintubation	3 (0.7)	10 (3.8)	0 (0.0)	8 (6.6)	36 (1.3)	<0.001 *
Ventilator requirement	8 (1.7)	12 (4.5)	0 (0.0)	10 (8.3)	40 (1.5)	<0.001 *
PE	16 (3.5)	11 (4.1)	1 (2.7)	2 (1.7)	36 (1.3)	0.001 *
Renal insufficiency	5 (1.2)	6 (2.5)	0 (0.0)	0 (0.0)	12 (0.5)	0.004 *
ARF	1 (0.2)	2 (0.8)	0 (0.0)	1 (0.8)	3 (0.1)	0.093
UTI	22 (4.8)	15 (5.6)	2 (5.4)	4 (3.3)	62 (2.3)	0.001 *
Cardiac arrest or MI	3 (0.7)	2 (0.8)	0 (0.0)	2 (1.7)	10 (0.4)	0.260
DVT	7 (1.5)	9 (3.4)	0 (0.0)	2 (1.7)	30 (1.1)	0.032 *
*C. diff* colitis	0 (0.0)	3 (1.5)	0 (0.0)	1 (1.2)	6 (0.3)	0.039 *
Systemic sepsis	12 (2.6)	8 (3.0)	1 (2.7)	4 (3.3)	53 (1.9)	0.576
Septic shock	2 (0.4)	2 (0.8)	1 (2.7)	5 (4.1)	9 (0.3)	<0.001 *
Postoperative RBC transfusion	174 (37.8)	145 (54.5)	12 (32.4)	64 (52.9)	1143 (41.5)	<0.001 *
AE severity, n (%)						
MAE	42 (9.1)	38 (14.3)	4 (10.8)	11 (9.1)	166 (6.0)	<0.001 *
SAE	197 (42.8)	156 (58.6)	13 (35.1)	70 (57.9)	1221 (44.3)	<0.001 *
Hospital length of stay (days), mean (SD)	6.41 (5.09)	8.92 (7.58)	12.61 (11.30)	13.49 (12.72)	6.50 (6.24)	<0.001 *
Total operation time (hours), mean (SD)	5.49 (2.69)	5.41 (2.76)	4.64 (2.67)	4.80 (2.29)	5.51 (2.62)	0.017 *
Readmission, n (%)	40 (8.7)	28 (10.6)	3 (8.1)	14 (11.6)	171 (6.3)	0.010 *
Reoperation, n (%)	26 (5.7)	16 (6.0)	4 (10.8)	8 (6.6)	143 (5.2)	0.568
Non-routine discharge, n (%)	210 (46.2)	154 (58.6)	28 (77.8)	79 (65.8)	587 (21.4)	<0.001 *
Mortality, n (%)	3 (0.7)	4 (1.5)	1 (2.7)	9 (7.4)	9 (0.3)	<0.001 *

AE: adverse event; SSI: surgical site infection; PNA: pneumonia; PE: pulmonary embolism; ARF: acute renal failure; UTI: urinary tract infection; MI: myocardial infarction; DVT: deep vein thrombosis; *C. diff*: *Clostridioides difficile*; RBC: red blood cell; MAE: minor adverse event; SAE: severe adverse event; SD: standard deviation; * Statistically significant, unadjusted *p*-value.

**Table 3:** Multivariable logistic regression model on the odds of extended hospital length of stay and 30-day adverse events.

	Adjusted OR	Lower Limit 95% CI	Upper Limit 95% CI	*p*-Value
**Extended Hospital Length of Stay**				
RAI-rev	1.03	1.01	1.04	<0.001 *
Anemic	1.84	1.45	2.35	<0.001 *
Malnourished	2.34	1.69	3.24	<0.001 *
Female	1.39	1.10	1.75	0.006 *
ASA				
1–2	REF	REF	REF	REF
≥3	1.90	1.47	2.47	<0.001 *
Dependent functional status	2.05	1.39	3.03	<0.001 *
Bleeding disorder	1.68	0.91	3.12	0.098
**30-Day Adverse Events**				
RAI-rev	1.07	1.04	1.11	<0.001 *
Anemic	2.09	1.66	2.61	<0.001 *
Malnourished	0.93	0.67	1.28	0.646
Age	0.98	0.96	0.99	0.001 *
Female	2.17	1.72	2.75	<0.001 *
Race/Ethnicity				
NHW	REF	REF	REF	REF
NHB	0.66	0.48	0.91	0.012 *
Hispanic	0.67	0.44	1.02	0.061
Other	1.25	0.74	2.09	0.400
ASA				
1–2	REF	REF	REF	REF
≥ 3	1.54	1.24	1.91	<0.001 *
Diabetes mellitus	0.81	0.62	1.06	0.123
Disseminated cancer	0.28	0.12	0.62	0.002 *
Smoking	0.60	0.47	0.77	<0.001 *

OR: odds ratio; CI: confidence interval; RAI-rev: revised risk analysis index; ASA: American Society of Anesthesiologists classification; NHW: non-Hispanic White; NHB: non-Hispanic Black; * Statistically significant, unadjusted *p*-value.

**Table 4:** Multivariable logistic regression model on the odds of nonroutine discharge and 30-day mortality.

	Adjusted OR	Lower Limit 95% CI	Upper Limit 95% CI	*p*-Value
**Nonroutine Discharge**				
RAI-rev	1.11	1.06	1.16	<0.001 *
Anemic	1.25	0.97	1.62	0.090
Malnourished	1.57	1.08	2.29	0.018 *
Age	1.03	1.01	1.05	<0.001 *
Female	1.45	1.09	1.92	0.010 *
BMI	1.02	1.00	1.04	0.034 *
Hypertension	0.62	0.48	0.80	<0.001 *
Diabetes mellitus	1.36	1.00	1.85	0.053
COPD	1.41	0.89	2.25	0.145
Disseminated cancer	0.19	0.07	0.49	<0.001 *
SAE	1.65	1.28	2.14	<0.001 *
LOS (days)	1.09	1.07	1.12	<0.001 *
Operation time (hours)	1.14	1.08	1.19	<0.001 *
**30-Day Mortality**				
RAI-rev	1.10	1.04	1.17	0.001 *
Anemic	1.04	0.34	3.17	0.951
Malnourished	3.79	1.24	11.60	0.020 *
Age	0.97	0.94	1.00	0.081
Electrolyte abnormality	2.54	0.83	7.78	0.102
Smoking	0.25	0.03	2.07	0.199
Pre-operative steroids	5.74	2.10	15.69	<0.001 *
Bleeding disorder	3.19	0.75	13.60	0.117
MAE	6.44	2.47	16.80	<0.001 *
SAE	2.94	0.93	9.37	0.068

OR: odds ratio; CI: confidence interval; RAI-rev: revised risk analysis index; BMI: body mass index; COPD: chronic obstructive pulmonary disease; SAE: severe adverse event; LOS: hospital length of stay; MAE: minor adverse event; * Statistically significant, unadjusted *p*-value.

## Data Availability

Data were obtained from the ACS NSQIP and are available from the authors with the permission of ACS.

## Supplementary Materials

The following supporting information can be downloaded at: https://www.mdpi.com/article/10.3390/jcm14155379/s1, Table S1. List of International Classification of Disease codes used to identify spinal deformity patients; Supplementary Table S2: Score assignments for the revised Risk Analysis Index (RAI-rev); Table S3: Observations with Missing Data.

## Abbreviations

The following abbreviations are used in this manuscript:

ASD	Adult Spinal Deformity
ACS	American College of Surgeons
NSQIP	National Surgical Quality Improvement Program
PSF	Posterior Spinal Fusion
ICD	International Classification of Disease
CPT	Current Procedural Terminology
RAI-rev	Revised Risk Analysis Index
ROC	Receiver Operating Characteristic
LOS	Length of Stay
AE	Adverse Event
NRD	Non-routine Discharge
OR	Odds Ratio
CI	Confidence Interval
CHF	Congestive Heart Failure
BMI	Body Mass Index
ASA	American Society of Anesthesiologists
COPD	Chronic Obstructive Pulmonary Disease
SSI	Surgical Site Infection
PNA	Pneumonia
PE	Pulmonary Embolism
ARF	Acute Renal Failure
UTI	Urinary Tract Infection
MI	Myocardial Infarction
DVT	Deep Vein Thrombosis
RBC	Red Blood Cell
MAE	Minor Adverse Event
SAE	Severe Adverse Event
AIC	Akaike Information Criterion
AUC	Area Under the Curve
F	Frail Alone
FA	Frail + Anemic
FM	Frail + Malnourished
FAM	Frail + Anemic + Malnourished
NF	Not Frail
ERAS	Enhanced Recovery After Surgery
