# Supplementary material for: Composite RAI, Malnutrition, and Anemia Model Superiorly Predicts 30-Day Morbidity and Mortality After Surgery for Adult Spinal Deformity
Source: J Clin Med. 2025 Jul 30;14(15):5379. doi: 10.3390/jcm14155379 (PMC12347132; doi:10.3390/jcm14155379)
Supplement: Supplementary file 1 [file jcm-14-05379-s001.zip › JCM_ASD_RAI_Malnutrition_Anemia_Supplementary Table S1.pdf]

Table S1. List of International Classification of Disease codes used to identify spinal deformity patients.

ICD Revision	Codes
ICD-9	737.00, 737.10, 737.11, 737.12, 737.19, 737.30, 737.31, 737.32, 737.33, 737.34, 737.39, 737.40, 737.41, 737.42, 737.43, 737.8, 756.19
ICD-10	M40.00, M40.03, M40.04, M40.05, M40.12, M40.13, M40.14, M40.30, M40.35, M40.36, M40.37, M40.50, M40.55, M40.56, M40.57, M40.292, M40.293, M40.294, M40.295, M40.299, M41.00, M41.02, M41.03, M41.04, M41.05, M41.06, M41.07, M41.08, M41.112, M41.113, M41.114, M41.115, M41.116, M41.117, M41.119, M41.122, M41.123, M41.124, M41.125, M41.126, M41.127, M41.129, M41.20, M41.22, M41.23, M41.24, M41.25, M41.26, M41.27, M41.30, M41.34, M41.35, M41.40, M41.41, M41.42, M41.43, M41.44, M41.45, M41.46, M41.47, M41.50, M41.52, M41.53, M41.54, M41.55, M41.56, M41.57, M41.80, M41.82, M41.83, M41.84, M41.85, M41.86, M41.87, M41.9, M43.8X1, M43.8X2, M43.8X3, M43.8X4, M43.8X5, M43.8X6, M43.8X7, M43.8X8, M43.8X9, M43.9, Q76.411, Q76.412, Q76.413, Q76.414, Q76.415, Q76.419, Q76.49

ICD: International Classification of Disease
