# Supplementary material for: Composite RAI, Malnutrition, and Anemia Model Superiorly Predicts 30-Day Morbidity and Mortality After Surgery for Adult Spinal Deformity
Source: J Clin Med. 2025 Jul 30;14(15):5379. doi: 10.3390/jcm14155379 (PMC12347132; doi:10.3390/jcm14155379)
Supplement: Supplementary file 1 [file jcm-14-05379-s001.zip › JCM_ASD_RAI_Malnutrition_Anemia_Supplementary Table S2.pdf]

Supplementary Table S2: Score assignments for the revised Risk Analysis Index (RAI-rev), adapted from Conlon et al.

**Variable(s)**	**RAI-rev score**
Male	+3
**Age and Cancer**	
Age <20 with cancer	+28
Age 20-24 with cancer	+29
Age 25-29 with cancer	+29
Age 30-34 with cancer	+30
Age 35-39 with cancer	+30
Age 40-44 with cancer	+31
Age 45-49 with cancer	+31
Age 50-54 with cancer	+32
Age 55-59 with cancer	+32
Age 60-64 with cancer	+33
Age 65-69 with cancer	+34
Age 70-74 with cancer	+34
Age 75-79 with cancer	+35
Age 80-84 with cancer	+35
Age 85-89 with cancer	+36
Age <20 without cancer	+0
Age 20-24 without cancer	+1
Age 25-29 without cancer	+4
Age 30-34 without cancer	+6
Age 35-39 without cancer	+8
Age 40-44 without cancer	+10
Age 45-49 without cancer	+12
Age 50-54 without cancer	+14
Age 55-59 without cancer	+16
Age 60-64 without cancer	+18
Age 65-69 without cancer	+20
Age 70-74 without cancer	+22
Age 75-79 without cancer	+24
Age 80-84 without cancer	+26
Age 85-89 without cancer	+28
Cancer diagnosis	N/A
Chronic kidney disease (end stage or dialysis)	+8
Congestive heart failure	+5
Dyspnea at rest	+3
Poor appetite (weight loss as proxy)	+4
Unintentional weight loss	+4
Residence at high level of care facility (transferred from non-home or intermediate care unit)	+1
**Functional status**	
Functional dependent status (partial)	+7
Functional dependent status (total)	+14
