# Supplementary material for: Composite RAI, Malnutrition, and Anemia Model Superiorly Predicts 30-Day Morbidity and Mortality After Surgery for Adult Spinal Deformity
Source: J Clin Med. 2025 Jul 30;14(15):5379. doi: 10.3390/jcm14155379 (PMC12347132; doi:10.3390/jcm14155379)
Supplement: Supplementary file 1 [file jcm-14-05379-s001.zip › JCM_ASD_RAI_Malnutrition_Anemia_Supplementary Table S3.pdf]

Supplementary Table S3: Observations with Missing Data

Variable	Missing count
Race/ethnicity	308
BMI	18
ASA classification	6
Dependent functional status	12
Electrolyte abnormality	341
Hospital length of stay	27
Operation time	1
Nonroutine discharge	26

ASA: American Society of Anesthesiologists classification; BMI: Body Mass Index
